# Oleuropein in Olive and its Pharmacological Effects

**DOI:** 10.3797/scipharm.0912-18

**Published:** 2010-04-23

**Authors:** Syed Haris Omar

**Affiliations:** College of Pharmacy, Qassim University, P.O. Box-31922, Buraidah-51418, Saudi Arabia

**Keywords:** Mediterranean diet, Olive, Phenolic compound, Oleuropein

## Abstract

Olive from *Olea europaea* is native to the Mediterranean region and, both the oil and the fruit are some of the main components of the Mediterranean diet. The main active constituents of olive oil include oleic acid, phenolic constituents, and squalene. The main phenolic compounds, hydroxytyrosol and oleuropein, give extra-virgin olive oil its bitter, pungent taste. The present review focuses on recent works that have analyzed the relationship between the major phenolic compound oleuropein and its pharmacological activities including antioxidant, anti-inflammatory, anti-atherogenic, anti-cancer activities, antimicrobial activity, antiviral activity, hypolipidemic and hypoglycemic effect.

## 1. Introduction

Several species within the olive family, botanically known as *Olea europaea*, provide commercial products such as food, lumber, cosmetics and medicine. Olive oil is a component of the Mediterranean diet, containing variable amounts of triacylglycerols and small quantities of free fatty acids, glycerol, pigments, aroma compounds, sterols, tocopherols, phenols, unidentified resinous components and others [1]. The pharmacological properties of olive oil, the olive fruit and its leaves have been recognized as important components of medicine and a healthy diet because of their phenolic content [2].

## 2. Occurrence

**Fig. 1.**
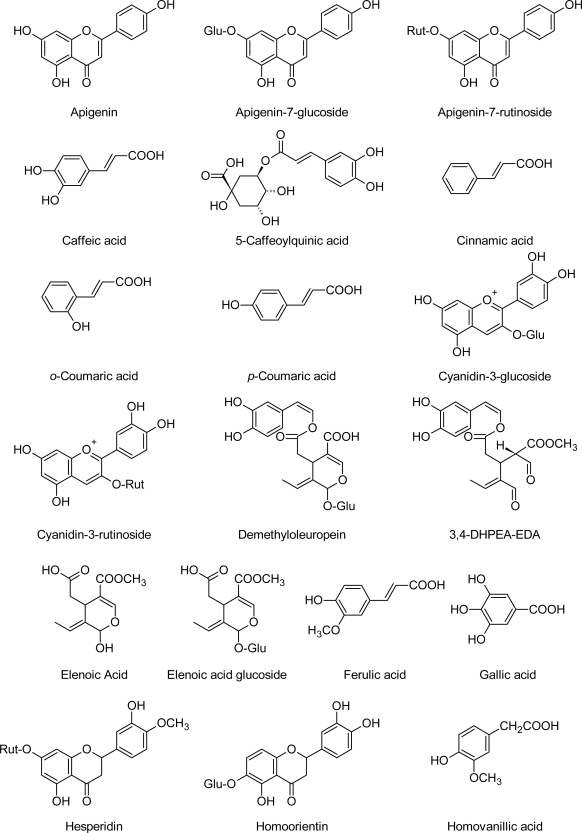

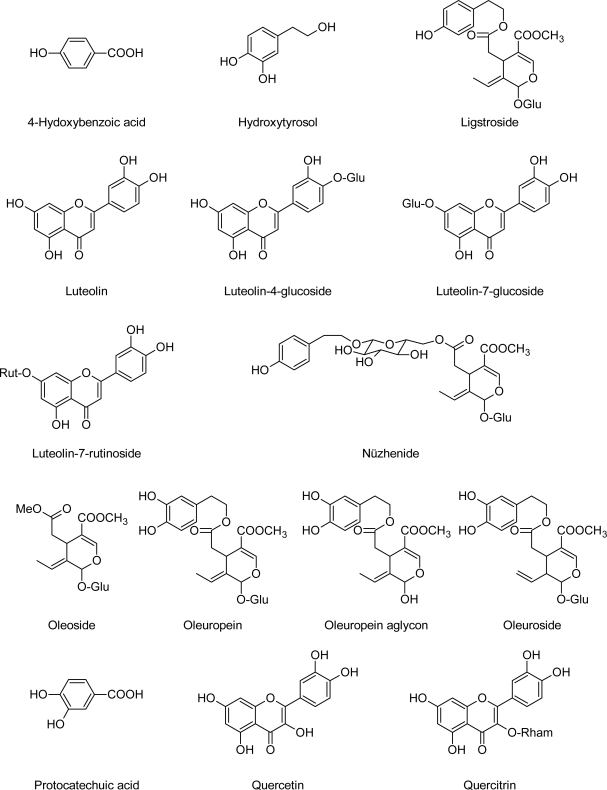

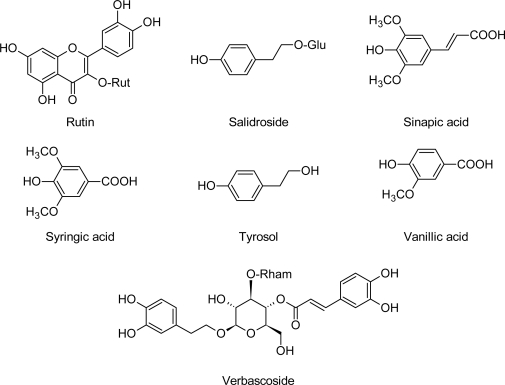
Alphabetical list of structures of phenolic compounds.

Phenolic compounds are found in all parts of the olive plant, but their nature and concentration varies greatly between the various tissues (Fig. 1). In *Olea europaea*, oleuropein, demethyl-oleuropein, ligstroside, and oleoside represent the predominant phenolic oleosides [3], whereas verbascoside [4] is the main hydroxycinnamic derivative of the olive fruit [5]. Oleuropein is generally the most prominent phenolic compound in olive cultivars and can reach concentrations of up to 140 mg g^−1^ on a dry matter basis in young olives [6] and 60–90 mg g^−1^ of dry matter in the leaves [7].

Various methods have been developed to analyze the qualitative and quantitative occurrence of phenolic and secoiridoid compounds: the simple techniques include Thin Layer Chromatography (TLC) [8], reversed phase High Performance Liquid Chromatography (HPLC) [9, 10], Gas Chromatography-Mass Spectrometry (GC-MS) [11] and Capillary Electrophoresis (CE) [12]; and the sophisticated methods involve Full Automatic Modeling System (FAMS) or Tetramethylsilane NMR [13], Electrospray Ionization Tandem Mass Spectrometry (ESI-MS/MS) and High-Resolution Measurements (HRMS) [14] LC/SPE/NMR, 2D-NMR, MALDI-TQF-MS [15–18]. In the fruits, phenyl acids, flavonoids and secoiridoids have been reported, with phenolic compounds representing 1–3% (w/v) of the olive [19]. In the leaves, oleuropein makes up 19% (w/w) and flavonoids make up 1.8% (w/w), of which 0.8% is luteolin 7-glucoside [7].

Oleuropein not only occurs in the *Olea* genus, but also occurs in many other genera belonging to the *Oleaceae* family and has been previously described in *Fraxinus excelsior, F. angustifolia, F. chinensis, Syringa josikaea, S. vulgaris, Philyrea latifolia, Ligustrum ovalifolium, L. vulgare*, and many others [3].

## 3. Chemistry, biosynthesis and fate of oleuropein

### 3.1. Chemistry

**Fig. 2.**
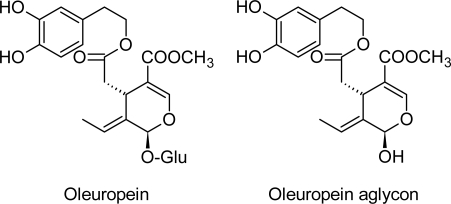
Structures of oleuropein and oleuropein aglycone

Oleuropein belongs to the secoiridoids, which are abundant in *Oleaceae, Gentianaceae, Cornaleae,* as well as many other plants. Iridoids and secoiridoids are compounds that are usually glycosidically bound and are produced from the secondary metabolism of terpenes as precursors of various indole alkaloids. The secoiridoids in *Oleaceae* are usually derived from the oleoside type of glucosides (oleosides), which are characterized by an exocyclic 8,9-olefinic functionality, a combination of elenolic acid and a glucosidic residue. Oleuropein is an ester of 2-(3,4-dihydroxyphenyl)ethanol (hydroxytyrosol) and has the oleosidic skeleton that is common to the secoiridoid glucosides of *Oleaceae* [3], mainly in its aglycone form, which makes the sugar moiety insoluble in oil (Fig. 2).

### 3.2. Biosynthesis of oleuropein

**Fig. 3.**
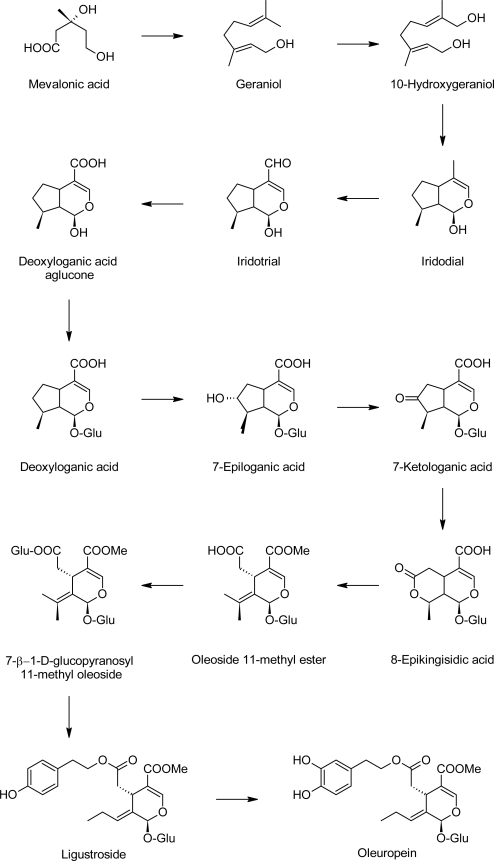
Proposed biosynthetic pathway for oleuropein in *Oleaceae*

The biosynthesis of oleuropein in *Oleaceae* proceeds via a branching in the mevalonic acid pathway from the secondary metabolism, resulting in the formation of oleosides [20]. From these compounds, secoiridoids are derived [21]. The biosynthesis of oleosides is similar to that of secologanin-derived secoiridoids in Gentianales and Cornales. In these compounds, the carbon skeleton is derived from mevalonic acid. Geraniol, 10-hydroxygeranoil as well as 10-hydroxynerol and iridoidal are known precursors of loganin. Later, deoxyloganic acid, 7-epiloganic acid and loganic acid are incorporated into ligustroside, a direct precursor of oleuropein, via a 7-ketologanic acid intermediate. The sequences of the steps between deoxyloganic acid and 7-ketologanin may differ between plant species and times of the year [22]. In *O. europaea*, both possible epoxides of secologanin and secoxyloganin can be precursors for oleuropein [21]. A plausible biosynthetic route from deoxyloganic acid, 7-epiloganic acid, 7-ketologanic acid, 8-epikingisidic acid, oleoside 11-methyl ester, 7-β-1-D-glucopyranosyl 11-methyl oleoside and ligustroside to oleuropein was proposed by Damtoft et al. [21] for *Oleaceae* (Fig. 3).

### 3.3. Fate of oleuropein

In the development of the olive fruit, three phases are usually distinguished: a growth phase, during which accumulation of oleuropein occurs; a green maturation phase that coincides with a reduction in the levels of chlorophyll and oleuropein; and a black maturation phase that is characterized by the appearance of anthocyanins and during which the oleuropein levels continue to fall [23]. Therefore, oleuropein is very abundant in the early stages: in young fruits, it can reach 14% of dry matter. Although lower, its level is still very important at harvest for green picked cultivars [24]. In black cultivars, its level declines rapidly during maturation [25]; in some varieties (*Oeuropaea* var leccino), it can even fall to zero when the fruits are completely black [26]. Elenolic acid glucoside and demethyloleuropein, glucosylated derivatives of oleuropein, appear at the beginning of green maturation as the oleuropein levels decline. Then, they accumulate, reaching their maximum during black maturation, until demethyloleuropein becomes the major constituent of black olives [26]. It is possible that these two compounds are formed from oleuropein by the action of esterases because esterase activity increases considerably during the first phase of maturation and reaches a maximum during black maturation [23]. The fruit of *Olea europaea* appears to accumulate only glucosylated derivatives of oleuropein. In contrast, dihydroxytyrosol and non-glucosylated secoiridoids derived from oleuropein have been found in the leaves [23, 27]. The decline in oleuropein also coincides with the decline in other quantitatively less important oleosides such as ligustroside and the increase in other phenolic compounds such as certain flavonoids and verbascoside [23]. In small, young olives, verbascoside is present only in traces, while ligustroside and cornoside are relatively abundant. When green olives reach normal size, the ligustroside disappears, and cornoside follows the same trend as the other compounds, easily transforming into halleridone [26].

Considerable differences in the content of tyrosol, hydroxytyrosol and tyrosol glucoside have also been found in the fruits during growth and ripening of the drupe [28, 29]; the increase in their levels consistently correlates with hydrolysis of the components with higher molecular weights [30]. The elenolic acid glucoside and hydroxytyrosol contents can be considered indicators for the maturation of olives [31]. Because of its interaction with a diphenol oxidase (PPO; EC 1.10.3.2), oleuropein is also involved in the browning that occurs in green table olives either after impact and wounding during harvesting or during subsequent processing treatments. Initially, this PPO is associated with the chloroplast membranes but becomes increasingly soluble during maturation.

Therefore, the degree of browning varies considerably depending on the physiological stage of the fruit. Browning was found to correlate with the oleuropein content and not with PPO activity, indicating that endogenous substrates are the main limiting factor [32].

## 4. Bioavailability of oleuropein

Phenolic compounds from virgin olive oil have been demonstrated to be highly bioavailable. Vissers et al. found that absorption of administered ligistroside-aglycone, hydroxytyrosol, tyrosol and oleuropein-aglycone was 55–60% in human subjects [33]. They also suggested that an important step in the metabolism of olive oil phenolics oleuropein-glycoside to oleuropein ad ligistroside-aglycones is their formation into hydroxytyrosol or tyrosol [33]. This hypothesis was supported by their finding that 15% of an oleuropein-glycoside supplement administered to healthy human subjects was excreted in urine as hydroxytyrosol and tyrosol [33]. Another two studies showed that oleuropein is rapidly absorbed after oral administration with a maximum plasma concentration occurring 2 h after administration. Hydroxytyrosol was its most important metabolite. Both compounds are rapidly distributed and excreted in urine mainly as glucoronides or in very low concentrations as free forms [34, 35]. Furthermore, the mechanism of absorption of olive oil phenolics remains unclear.

## 5. Oleuropein and Health

**Fig. 4.**
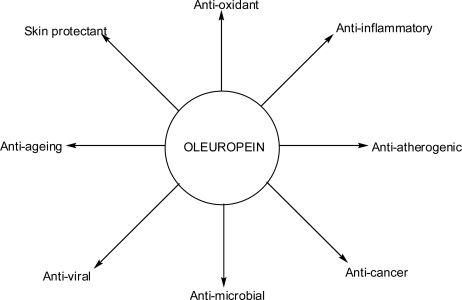
Pharmacological effects of oleuropein

Oleuropein has several pharmacological properties (Fig. 4), including antioxidant [2], anti-inflammatory [36], anti-atherogenic [37], anti-cancer [38], antimicrobial [39], and antiviral [40], and for these reasons, it is commercially available as food supplement in Mediterranean countries. In addition, oleuropein has been shown to be cardioprotective against acute adriamycin cardiotoxicity [41] and has been shown to exhibit anti-ischemic and hypolipidemic activities [42].

### 5.1. Antioxidant activity

Oleuropein potently and dose-dependently inhibits copper sulphate-induced oxidation of low-density lipoproteins (LDL) [43, 44]. According to De la Puerta et al. [45], oleuropein has both the ability to scavenge nitric oxide and to cause an increase in the inducible nitric oxide synthase (iNOS) expression in the cell. A scavenging effect of oleuropein was demonstrated with respect to hypochlorous acid (HOCl) [43]. HOCl is an oxidative substance produced *in vivo* by neutrophil myeloperoxidase at the site of inflammation and can cause damage to proteins including enzymes.

Coni et al. [46] conducted a study with laboratory rabbits fed special diets that contained olive oil and oleuropein. The results indicate that the addition of oleuropein increases the ability of LDL to resist oxidation and at the same time reduces the plasma levels of total, free, and esterified cholesterol.

Additionally, the potential protective effects of oleuropein have been investigated in isolated rat hearts by Manna et al. [47]. The organs were subjected to 30 min of no-flow global ischemia and then reperfused. At different intervals, the coronary heart effluent was collected and assayed for creatine kinase activity and reduced and oxidized glutathione. The extent of lipid peroxidation was evaluated by measuring the concentration of thiobarbituric acid-reactive substance in the muscle. According to the authors, the findings of the study strengthen the hypothesis that the health benefits of olive oil are related to the oleuropein derivatives that are present in olive oil. De la Puerta et al. [48] determined the anti-eicosanoid and antioxidant effects in leukocytes of the principal phenolic compounds (oleuropein, tyrosol, hydroxytyrosol and caffeic acid) from the polar fraction of olive oil. Moreover, Visioli et al. [49] demonstrated that the administration of catecholic phenolic from olive oil (oleuropein) dose-dependently decreases the urinary excretion of 8-iso-PGF2α, indicating lower *in vivo* lipid peroxidation in supplemented volunteers.

### 5.2. Anti-inflammatory effect

Visioli et al. [36] showed that oleuropein increases nitric oxide (NO) production in macrophages challenged with lipopolysaccharide through induction of the inducible form of the enzyme nitric oxide synthase, thus increasing the functional activity of these immunocompetent cells. It is well known that oleuropein elicits anti-inflammatory effects by inhibiting lypoxygenase activity and the production of leukotriene B_4_ [48].

### 5.3. Anti-atherogenic effect

Visioli and Galli reported that Oleuropein shows anti-atherogenic activity [50]. In 2003, Carluccio MA et al. [51] reported that oleuropein reduces monocytoid cell adhesion to stimulated endothelium as well as vascular cell adhesion molecule-1 (VCAM-1) mRNA and protein. Reflow in ischemic hearts was accompanied by a prompt release of oxidized glutathione; in ischemic hearts pretreated with oleuropein, this release was significantly reduced and was accompanied by prevention of membrane lipid peroxidation, which is considered a key factor in the pathogenesis of atherosclerosis [47].

### 5.4. Anti-cancer effect

A plethora of minor constituents in olive oil have been identified as effective agents in mitigating the initiation, promotion and progression of multistage carcinogenesis.

Hamdi and Castellon showed that oleuropein inhibits growth of LN-18 cells, a poorly differentiated glioblastoma cell line; TF-1a, a erythroleukemia; and tumor cell lines derived from advanced-grade human tumors (786-O, renal cell adenocarcinoma; T-47D, infiltrating ductal carcinoma of the breast pleural effusion; RPMI-7951, malignant melanoma of the skin-lymph node metastasis; and LoVo, colorectal adenocarcinoma cells) in Swiss albino mice with soft tissue sarcoma [52].

Menendez et al. [53] showed that oleuropein aglycone is the most potent phenolic compound in decreasing breast cancer cell viability. HER2 oncogene-amplified SKBR3 cells were ∼5-times more sensitive to oleuropein aglycone than HER2-negative MCF-7 cells.

Subsequently, Menendez et al. [54] showed that the secoiridoids deacetoxy oleuropein aglycone, ligstroside aglycone, and oleuropein aglycone, induce strong tumoricidal effects within a micromolar range by selectively triggering high levels of apoptotic cell death in HER2-overexpressing breast carcinomas. These compounds markedly depleted HER2 protein and reduced HER2 tyrosine autophosphorylation in a dose- and time-dependent manner [54].

Recently, Han et al. [55] reported that 200 lg/mL of oleuropein remarkably reduces the viability of MCF-7 cells and decreases the number of MCF-7 cells by inhibiting the rate of cell proliferation and inducing cell apoptosis. Additionally, oleuropein exhibited a statistically significant block of G_1_ to S phase transition, which was manifested by the increase in the number of cells in the G0/G_1_ phase [55].

Goulas et al. [56] demonstrated the antiproliferative activity of crude extracts and phytochemicals (the dominant compound of the extracts is oleuropein) against cell lines at low micromolar concentrations. These extracts inhibit cell proliferation of human breast adenocarcinoma (MCF-7), human urinary bladder carcinoma (T-24) and bovine brain capillary endothelial (BBCE).

### 5.5. Antimicrobial effect

Oleuropein has been shown to have strong antimicrobial activity against both Gram-negative and Gram-positive bacteria [57–59] as well as mycoplasma [60]. Phenolic structures similar to oleuropein seem to produce its antibacterial effect by damaging the bacterial membrane and/or disrupting cell peptidoglycans. Different authors have used biophysical assays to study the interaction between oleuropein and membrane lipids [61]; however, the exact mechanism of the antimicrobial activity of oleuropein is still not completely established, although some authors have proposed that it is due to the presence of the ortho-diphenolic system (catechol) [57]. In 2001, Saija and Uccella [62] proposed that the glycoside group modifies the ability to penetrate the cell membrane and get to the target site. Effective interference with the production procedures of certain amino acids necessary for the growth of specific microorganisms has also been suggested. Another mechanism proposed is the direct stimulation of phagocytosis as a response of the immune system to microbes of all types.

Oleuropein and hydrolysis products are able to inhibit the development and production of enterotoxin B by *Staphylococcus aureus*, the development of *Salmonella enteritidis* and the germination and consequent development of spores of *Bacillus cereus* [57–67]. Oleuropein and other phenolic compounds (p-hydroxybenzoic, vanillic and p-coumaric acids) completely inhibit the development of *Klebsiella pneumoniae, Escherichia coli* and *B. cereus* [58].

Recently, Sudjana et al. [68] showed the antimicrobial activity of commercial *Olea europaea* (olive) leaf extracts (abundantly oleuropein) against *Campylobacter jejuni, Helicobacter pylori* and methicillin-resistant *Staphylococcus aureus* (MRSA). The authors also demonstrated these extracts play a role in regulating the composition of the gastric flora by selectively reducing levels of *H. pylori* and *C. jejuni*.

### 5.6. Antiviral effect

In a U.S. patent, it has been claimed that oleuropein has potent antiviral activities against herpes mononucleosis, hepatitis virus, rotavirus, bovine rhinovirus, canine parvovirus, and feline leukemia virus [69]. Studies have also shown that oleuropein exhibits a significant antiviral activity against respiratory syncytial virus and para-influenza type 3 virus [70].

There is also one anecdotal report that olive leaf extracts augment the activity of the HIV-RT inhibitor 3TC [71]. The olive leaf extracts were investigated for their antiviral activity against viral hemorrhagic septicemia virus (VHSV), a salmonid rhabdovirus, and against HIV-1 infection and replication [72]. Cell-to-cell transmission of HIV was inhibited in a dose-dependent manner with EC_50_s of 0.2 μg/ml, and HIV replication was inhibited in an *in vitro* experiment [73].

One of the suspected targets for olive leaf extract (mainly oleuropein) action is HIV-1 gp41 (surface glycoprotein subunit), which is responsible for HIV entry into normal cells. In order to establish HIV protein targets of olive leaf extract and its inhibitory action at the molecular level, Lee-Huang et al. [74] reported a joint theoretical and experimental effort has been carried out to help achieve this goal.

### 5.7 Skin protectant

Ancora et al. [75] have shown that the phenol components of olive oil have a direct antioxidant action on skin, especially oleuropein, which acts as a free radical scavenger at the skin level. Recently, Kimura and Sumiyoshi [76] suggested that the preventative effects of olive leaf extracts and oleuropein on chronic UVB-induced skin damage and carcinogenesis and tumor growth may be due to inhibition of the expression of VEGF, MMP-2, MMP-9, and MMP-13 through a reduction in COX-2 levels.

### 5.8 Anti-aging

Normal human fibroblasts undergo replicative senescence due to both genetic and environmental factors. The proteasome, a multicatalytic nonlysosomal protease, has impaired function during aging, while its increased expression delays senescence in human fibroblasts. Katsiki et al. [77] demonstrated that oleuropein enhances proteasome activities in vitro more effectively than other known chemical activators, possibly through conformational changes of the proteasome. Moreover, continuous treatment of early passage human embryonic fibroblasts with oleuropein decreases the intracellular levels of reactive oxygen species (ROS), reduces the amount of oxidized proteins through increased proteasome-mediated degradation rates and retains proteasome function during replicative senescence. Importantly, oleuropein-treated cultures exhibit a delay in the appearance of senescence morphology, and their life span is extended by approximately 15% [77].

### 5.9 Neuroprotective activity

**Fig. 5.**
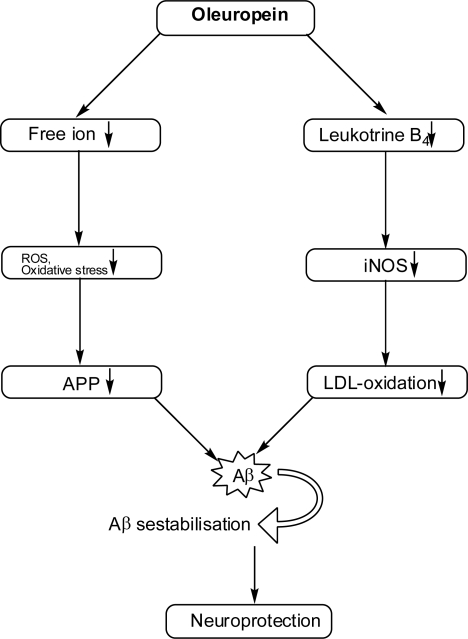
Diagrammatic representation of the neuroprotective role of oleuropein

According to the free radical theory, aging is the result of oxidative injury, mainly to mitochondria, over the lifetime of an individual. Some of the oxidative damage cannot be entirely counteracted, which leads to cellular dysfunction. Mitochondrial membranes are very sensitive to free radical attack because of the presence of a double bond carbon-carbon in the lipid tails of its phospholipids, which leads to the production of cognitive and neurodegenerative disease. In vitro [78] and epidemiological [79] studies have pointed out the positive impact of natural extracted polyphenols on the incidence of age-related disorders, such as dementia. One study [80] has reported that oleuropein decreases or even prevents Aβ aggregation, which is inherent to Alzheimer’s disease (AD). The potential effect of oleuropein on brain function in AD is analogous to atherosclerosis because they both are age-dependent diseases in which abnormal accumulation of a normal metabolite (cholesterol and Aβ, respectively) precedes clinical symptoms and leads to disease [81,82]. The link between heart disease, hypercholesterolemia, and AD [83] is due to similar mechanisms of pathogenesis of these disorders. The circumstantial evidence that cholesterol-related interventions can alter Aβ deposition [84, 85] suggests that oleuropein might be promising in the management of AD. Furthermore, the importance of inflammatory processes in the clinical manifestation of AD [86, 87], combined with the epidemiological evidence of a protective effect of anti-inflammatory agents [88] against AD, suggest that a polyphenolic natural extract, such as oleuropein, could prove effective against age-dependent disease. The diagrammatic representation of the neuroprotective role of oleuropein is shown in Figure 5.

### 5.10 Other activity

Further pharmacological activity of oleuropein includes diverse healing properties due to its vasodilatory [89], anti-platelet aggregation [90], hypotensive [91, 92], anti-rheumatic [36], diuretic [91] and antipyretic [93] effects. Prevention of free radical formation by oleuropein occurs through its ability to chelate metal ions, such as Cu and Fe, which catalyze free radical generation reactions [94], and through its inhibitory effect on several inflammatory enzymes like lipoxygenases [48]. Previously, oleuropein was reported to have an anti-hyperglycemic effect in diabetic rats [95]. Oleuropein inhibits hyperglycemia and oxidative stress induced by diabetes, which suggests that administration of oleuropein is helpful in the prevention of diabetic complications associated with oxidative stress [96].

## 6. Conclusion

Oleuropein, the main glycoside present in olives, and hydroxytyrosol, the principal degradation product of oleuropein present in olive oil, have both been linked to reduction of coronary heart disease and certain cancers.

There are still numerous key issues that need to be answered, and these will require further research. Specifically, one question that remains unanswered is what the neuroprotective (Dementia, Parkinson’s, Alzheimer’s and Schizophrenia) roles of oleuropein are?
